# New design model for high efficiency cylindrical diffractive microlenses

**DOI:** 10.1038/s41598-017-14787-x

**Published:** 2017-11-27

**Authors:** Ye Li, Huan Zhao, Sheng-Fei Feng, Jia-Sheng Ye, Xin-Ke Wang, Wen-Feng Sun, Peng Han, Yan Zhang

**Affiliations:** Department of Physics, Capital Normal University, Beijing Key Laboratory of Metamaterials and Devices, Beijing Advanced Innovation Center for Imaging Technology, Key Laboratory of Terahertz Optoelectronics, Ministry of Education, Beijing, 100048 P. R. China

## Abstract

A new model, i.e., the decreasing thickness model (DTM) is proposed and employed for designing the cylindrical diffractive microlenses (CDMs). Focal performances of the designed CDMs are theoretically investigated by solving Maxwell’s equations with the boundary element method. For comparison, the CDMs designed by the traditional equal thickness model (ETM) are also studied. Theoretical simulations demonstrate that focal performances of the designed CDMs are improved a lot via replacing the traditional ETM with the proposed DTM. Concretely, the focal efficiency is heightened and the focal spot size is shrunk. Experimental measurements verify the theoretical simulations well. Especially, the above-mentioned improvements become more prominent for the CDM with a higher numerical aperture.

## Introduction

The diffractive microlens, as one of the most important components in micro optical systems, is widely used in beam focusing^1–4^ and object imaging^5–7^. Therefore, scientists make great efforts on its design^8–13^, performance analysis^14–18^, fabrication^19–22^ and practical applications^23–25^. In past literatures, the cylindrical diffractive microlens (CDM) is generally designed from a refractive microlens by removing the vertically abundant 2*π* phases^14,26^. Consequently, the CDM is a Fresnel lens with maximum phase of 2*π*. Since the Fresnel lens has equal maximum thicknesses in all the Fresnel zones, this design model is called as the equal thickness model (ETM).

If we reconsider the ETM more carefully, we will find its two drawbacks. Firstly, at the boundary jump point between two adjacent Fresnel zones, although the phase change is 2*π* along the optics axis direction, it deviates from 2*π* with respect to the preset focal position. Secondly, since different parts of the CDM boundary are segmented and downward shifted from a definite refractive microlens, they concentrate the incident plane wave on a series of sub focuses in the optics axis. As a result, at the preset focal position the field interference effect is destructed. Especially, if the CDM has a high numerical aperture, the destruction will be serious due to a large number of Fresnel zones^27,28^.

Aiming at the above two drawbacks, we propose a new model for designing the CDM by making two improvements. Firstly, the boundary jump points between two adjacent Fresnel zones are relocated, where the phase change is exactly 2*π* with respect to the preset focal position. Secondly, the CDM boundary equation is exactly deduced from Fermat’s principle, so that the incident plane wave inside a given Fresnel zone accumulates the same optical path. With this new design model, the CDM boundary can be determined. Since the maximum thickness monotonously decreases with the increase of the Fresnel zone ordinal number, we name it as the decreasing thickness model (DTM).

In order to verify the feasibility of the proposed DTM, we apply it to several design examples of the CDMs. Since the CDMs have very fine structures smaller than the wavelength, their focal performances are analyzed by solving Maxwell’s equations with the boundary element method (BEM)^14,29–31^. To show the superiority of the proposed DTM, the CDMs designed by the traditional ETM are also investigated. The characterized focal performances include focal efficiency, focal spot size and actual focal length. The focal efficiency is defined as the percentage ratio of the focused energy in the central lobe to the total incident energy. The focal spot size is defined as the dimension between the first lowest intensity positions on both sides of the central lobe in the actual focal plane. The actual focal length is the axial distance from the maximum intensity point to the origin point.

Moreover, the designed CDMs are fabricated by a photonic professional GT 3D laser lithography system^32^. Then, focal performances of the fabricated CDMs are experimentally measured. We expect for a significant improvement in experimental focal performances of the CDM designed by the DTM. If so, it will promote the application of the CDM to practical systems.

The layout of this paper is as follows. In the second part, we theoretically analyze and compare focal performances of the CDMs designed by the DTM and the ETM. Physical explanations are presented. In the third part, experimental fabrication and characterization of the CDMs are described in detail. The experimental measurements of the CDMs are compared with the theoretical simulations. In the fourth part, a concise conclusion is made. Finally, the design principle of the proposed DTM and the relevant formulas are delivered in the fifth part.

## Theoretical Performances of the Designed Cylindrical Diffractive Microlenses (CDMs)

**Figure 1:**
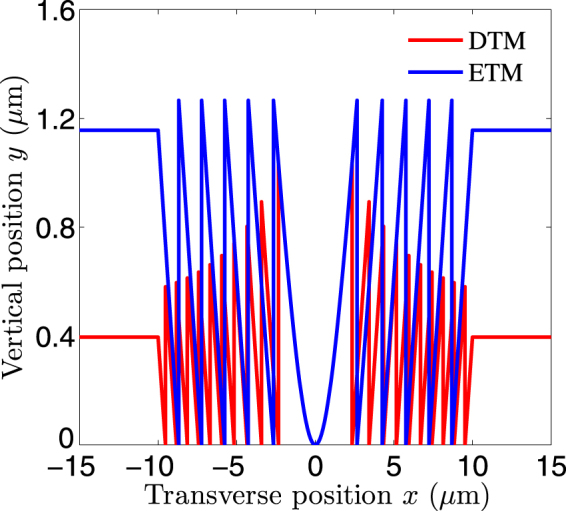
Boundary profiles of the cylindrical diffractive microlenses (CDMs) designed by the decreasing thickness model (DTM) and the equal thickness model (ETM). The red and blue curves correspond to the DTM and the ETM, respectively.

Detailed design principles and processes of the CDMs are described in Section 5 with formulas. On selecting a set of parameters, we can calculate the boundary profiles from Eqs (3) and (9) in Section 5 for the CDMs designed by the ETM (blue curve) and the DTM (red curve), respectively, as shown in Fig. 1. Apparently, the CDM designed by the ETM has the same maximum thicknesses in all the Fresnel zones, while the maximum thickness of the CDM designed by the DTM is gradually decreased. The selected parameters are as follows. The upper and lower regions are filled with glass and air, whose refractive indices^33^ are *n*_1_ = 1.5 and *n*_2_ = 1.0, respectively. The dual CDMs have the same diameters and the same preset focal lengths of *D* = 20 *μ*m and *f* = 4 *μ*m, respectively. The dual CDMs have the same *f*-numbers (*f*/# = *f*/*D*) of *f*/0.2. The incident wavelength is *λ* = 0.633 *μ*m. The incident plane wave is TE polarized, namely the electric field is along the infinite *z*-axis.

For the dual *f*/0.2 CDMs in Fig. 1, there exist three differences: the boundary jump position, the boundary curvature, and the maximum thickness. Locations of the boundary jump points are obtained from Eqs (5) and (7) in Section 5 for the CDMs designed by the ETM and the DTM, respectively. Since the boundaries of the dual CDMs are respectively calculated from Eqs (3) and (9) in Section 5, the boundary curvatures are different. Besides, the maximum thicknesses are different for the dual CDMs, due to different boundary equations and different jump positions.

**Figure 2:**
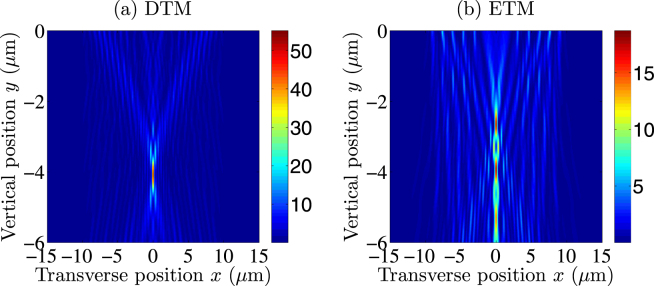
Focused intensity distributions for the dual *f*/0.2 CDMs designed by using (**a**) the DTM and (**b**) the ETM, respectively.

Next, focal performances of the dual *f*/0.2 CDMs are theoretically analyzed by the BEM, to show the superiority of the DTM to the ETM. Figure 2 displays the focused intensity distributions for the dual *f*/0.2 CDMs designed by the DTM and the ETM, respectively. The red and blue districts represent the regions with high and low intensities, respectively. It is seen from Fig. 2(a) that the CDM designed by the DTM has one focal spot with normalized peak intensity higher than 50. In contrast, it is seen from Fig. 2(b) that the CDM designed by the ETM has three axial focal spots with normalized peak intensity lower than 20. Physical explanations are as follows. For the CDM designed by the DTM, all the boundary points have the same phases or have a phase difference of 2*π* multiples with respect to the preset focal position, where a perfectly constructive interference effect is observed. However, for the CDM designed by the ETM, as the segmented boundaries are axially shifted from the refractive lens boundary, they generate a series of axial focal spots. It is concluded from Fig. 2 that the CDM designed by the DTM performs much better than that designed by the ETM.

**Figure 3:**
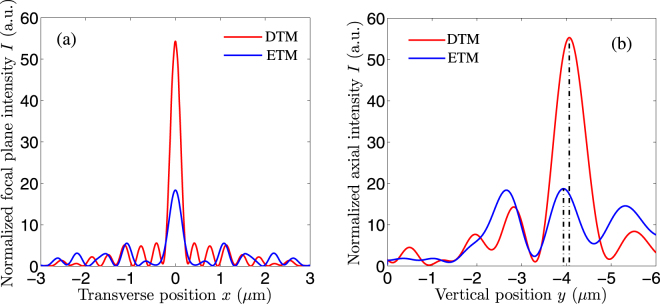
Normalized intensity distributions for the dual *f*/0.2 CDMs in (**a**) the actual focal plane and (**b**) the axial plane. The red and blue curves correspond to the CDMs designed by DTM and the ETM, respectively.

In order to compare focal performances of the dual CDMs more precisely, the normalized intensity distributions in the preset focal plane are plotted in Fig. 3(a). The red and blue curves correspond to the CDMs designed by the DTM and the ETM, respectively. Compared with the CDM designed by the ETM, the CDM designed by the DTM has a much higher peak intensity and a smaller focal spot size. Numerical results reveal that the normalized peak intensities are 54.3 and 18.4 for the CDMs designed by the DTM and the ETM, respectively. The corresponding focal efficiencies are 38.61% and 19.85%. The focal spot sizes are respectively 0.57 and 0.89 *μ*m. Figure 3(b) draws the normalized axial intensity distributions for the dual CDMs. The dashed lines mark the actual focal lengths. It is seen from Fig. 3(b) that the CDM designed by the DTM has a distinct focus, which situates around the preset focal position (−4 *μ*m). In contrast, the CDM designed by the ETM has three focuses. Numerical results reveal that the actual focal lengths are 4.06 and 3.93 *μ*m for the CDMs designed by the DTM and the ETM, respectively. Above all, the CDM designed by the DTM has a much higher focal efficiency, a smaller focal spot size and a more accurate focal length, in comparison with the CDM designed by the ETM. Namely, it is demonstrated again that the DTM is much superior to the ETM in the design of the CDM.

To furtherly show a general superiority of the DTM to the ETM in designing the CDMs, we select several *f*-numbers from 0.2 to 1.4 with an interval of 0.2. The preset focal lengths ranges from 4 to 28 *μ*m. Other parameters are the same as above. The theoretical focal performances of the designed CDMs are tabulated in Table 1. When the preset focal length is smaller than 20 *μ*m, focal efficiency for the DTM is much higher than that for the ETM. For large preset focal lengths, the focal efficiencies for the DTM and the ETM differ little since the two designed microlens profiles gradually coincide, according to Eqs (3) and (9) in Section 5. With the increase of the preset focal length, the focal spot size for the ETM is irregularly varied because the CDM boundaries in different Fresnel zones produce destructive interference at the preset focal position. In contrast, the focal spot size for the DTM monotonically increases with the increase of the preset focal length, since the CDM boundary is optimally designed based on the Fermat’s principle. For each preset focal length, the focal spot size for the DTM is smaller than that for the ETM. In addition, the CDM designed by the DTM has a more accurate focal length than that designed by the ETM. In conclusion, the DTM is much superior to the ETM for designing the CDMs, especially for small *f*-numbers.

**Table 1:** Theoretical focal performance comparison between the CDMs designed by the DTM and the ETM.

Preset focal length (*μ*m)	Focal efficiency (%)	Focal spot size (*μ*m)	Actual focal length (*μ*m)
Design Model	DTM ETM	DTM ETM	DTM ETM
4	38.61 19.85	0.57 0.89	4.06 3.93
8	48.19 32.39	0.85 1.79	8.11 10.19
12	55.72 42.85	1.07 1.98	11.94 15.54
16	64.51 52.24	1.27 1.99	15.83 18.95
20	69.38 65.03	1.51 2.37	19.69 22.19
24	73.10 73.38	1.74 3.11	23.35 25.73
28	75.66 73.46	1.98 2.11	27.28 29.12

## Experimental Focal Performances of the Fabricated CDMs

**Figure 4:**
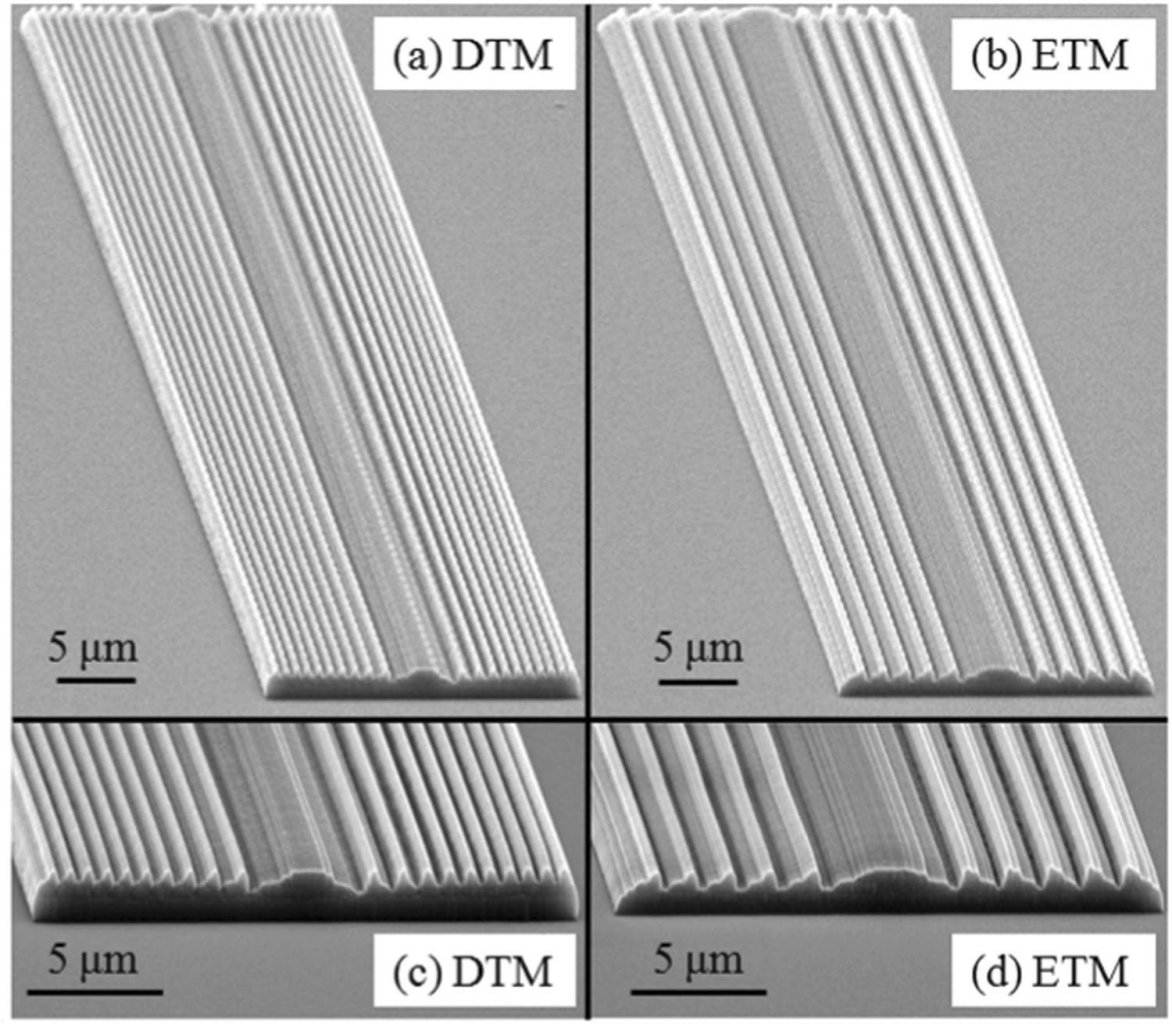
Scanning electron microscope images of the fabricated *f*/0.2 CDMs designed by the (**a**) DTM and the (**b**) ETM. (**c**,**d**) Are magnified cross-sectional images of (**a**,**b**) respectively.

The photonic professional GT 3D laser lithography system from the German Nanoscribe Company is utilized for fabrication of the above-designed CDMs, which provides a fast fabrication platform for arbitrarily complex three-dimensional nanostructures with extraordinary high resolution via two-photon polymerization^34–36^. Figure 4(a,b) show the scanning electron microscope (SEM) images of the fabricated CDMs designed by the DTM and the ETM, respectively. Figure 4(c,d) are magnified images of Fig. 4(a,b) for clearer observation of the cross-sectional profiles. The preset focal lengths of the fabricated microlenses are 4 *μ*m. In Fig. 4(a,b), the fabricated microlens profiles are overall uniform along the longitudinal direction. In Fig. 4(c,d), the fabricated ridges have sharp corners, being consistent with the designed profiles. It is also noted in Fig. 4(c,d) that there are some slight concaves on the cross-sectional profiles of the fabricated microlenses, because of instability during the system operation.

**Figure 5:**
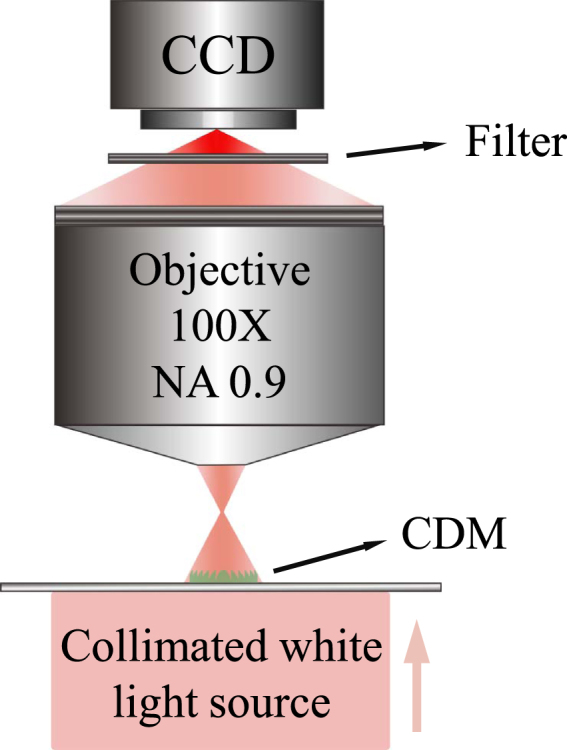
Schematic of the experimental system for measuring the focal performances of the fabricated CDMs.

Once the CDM fabrication is finished, we use the Olympus BX61 microscope to measure focal performances of the CDMs, as shown in Fig. 5. The collimated white light source is radiated from the build-in halogen lamp of the Olympus BX61 microscope. After passing through the CDM, it will be focused. A microscope objective (100×, NA = 0.9) is utilized to image the focal line on a charge-couple-device (CCD) camera. Since the CDM is designed at wavelength of 0.633 *μ*m, a single-band bandpass filter^37^ (Semrock FF01-631/4–25) is inserted in front of CCD camera. The filter transmission band ranges from 0.629 to 0.633 *μ*m with transmissivity higher than 90%.

**Figure 6:**
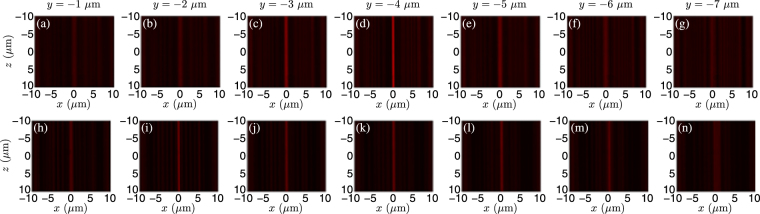
Measured intensities in several cross-sectional planes along the propagation direction. The upper row corresponds to the *f*/0.2 CDM designed by the DTM. (**a**–**g**) Correspond to the cross-sectional planes at *y* = −1 *μ*m to *y* = −7 *μ*m for every 1 *μ*m. The lower row is the same as the upper one except that the DTM is replaced by the ETM.

To see the dynamic focusing property of the fabricated CDMs, we measure the intensities in several cross-sectional planes along the propagation direction, as shown in Fig. 6. The upper row of Fig. 6 shows the measured intensities of the *f*/0.2 CDM designed by the DTM. Figure 6(a–g) correspond to the cross-sectional planes at *y* = −1 *μ*m to *y* = −7 *μ*m for every 1 *μ*m. From the upper row, the fabricated CDM designed by the DTM firstly converges and then diverges. The actual focal plane lies at *y* = −4 *μ*m. The lower row is the same as the upper one except that the DTM is replaced by the ETM. An obvious difference is that the CDM designed by the ETM has a much longer axial focal depth, ranging from *y* = −2 *μ*m to *y* = −5 *μ*m. The actual focal plane for the ETM situates at *y* = −2 *μ*m.

**Figure 7:**
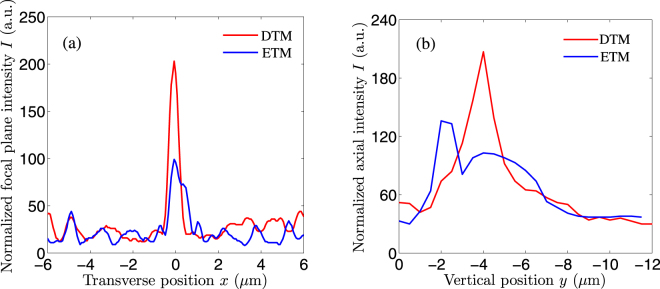
Measured intensity distributions for the fabricated *f*/0.2 CDMs in (**a**) the preset focal plane and (**b**) the axial plane. The red and blue curves correspond to the *f*/0.2 CDMs designed by the DTM and the ETM, respectively.

To see the normalized focal plane intensity more clearly, the experimental linescan intensity profiles of Fig. 6(d,k) along the *x*-axis are drawn in Fig. 7(a). The red and blue curves correspond to the CDMs designed by the DTM and the ETM, respectively. It is clearly seen from Fig. 7(a) that the CDM designed by the DTM has a higher peak intensity and a smaller focal spot size, compared with the CDM designed by the ETM. In experiment, the normalized peak intensities are 203 and 99 for the CDMs designed by the DTM and the ETM, respectively. The corresponding experimental focal efficiencies are 16.23% and 11.42%, and experimental focal spot sizes are 1.10 and 1.38 *μ*m. The differences between the experimental measurements and theoretical simulations can be explained as follows. The CDMs are theoretically designed to focus the incident plane wave to the preset focal position. However, the experimental SEM images show that the fabricated microlens profiles have slight concaves, which makes local curvature of the fabricated profile deviate from that of the designed profile. The fabricated profiles can no longer bend the incident plane wave to the preset focal position because of profile deviation, weakening the experimental focal performances. Accordingly, the experimental focal efficiency is decreased and the experimental focal spot size is increased, in comparison with the theoretical simulations.

We also extract the axial intensities from Fig. 6 and plot the normalized axial plane intensities, as shown in Fig. 7(b). The axial peak intensity for the DTM is much higher than that for the ETM. In addition, the experimental actual focal position is near to the preset one (−4 *μ*m) for the DTM, while it deviates away for the ETM. It can be explained in the following. For the CDM designed by the DTM, from Fig. 3(b) the theoretical axial intensity profile has a single sharp peak around −4 *μ*m. Although there exist some slight concaves in the fabricated microlens profile, the experimental actual focal length does not change. In contrast, for the CDM designed by the ETM, from Fig. 3(b) the theoretical axial intensity profile has three peaks. Especially, the two peaks around −2.5 and −4 *μ*m almost have identical intensity values. In this case, the fabrication errors of the microlens profile severely influence the measured axial intensities. That’s why the experimental actual focal length becomes 2.0 *μ*m for the fabricated CDM designed by the ETM (the axial step is 0.5 *μ*m in experiments).

Besides, we also fabricate the dual CDMs whose focal lengths are 6 *μ*m, i.e., the *f*-numbers are *f*/0.3. The experimental (theoretical) focal efficiencies are 20.13% (37.13%) and 16.99% (29.09%) for the *f*/0.3 CDMs designed by the DTM and the ETM, respectively. The corresponding experimental (theoretical) focal sizes are 1.37 (0.66) and 1.86 (1.38) *μ*m. The experimental (theoretical) actual focal lengths are 6.5 (6.12) and 8.0 (6.55) *μ*m for the *f*/0.3 CDMs designed by the DTM and the ETM, respectively. It is proved again in experiment that the CDM designed by the DTM has a higher focal efficiency, a smaller focal spot size and a more accurate focal length, compared with the CDM designed by the ETM.

## Conclusion

In this paper, the DTM is proposed for designing the CDMs based on Fermat’s principle without any approximation. Focal performances of the CDMs are theoretically analyzed by the BEM. The CDMs designed by the traditional ETM are also analyzed for comparison. Superiority of the DTM to the ETM is clearly demonstrated for designing the CDMs. This superiority becomes more notable, when the *f*-number is decreased. For instance, for the *f*/0.2 CDM, the focal efficiency is almost doubled and the focal spot size is reduced by one half. In addition, the designed CDMs are fabricated by the photonic professional GT 3D laser lithography system. Experimental focal performances of the fabricated CDMs agree well with the theoretical simulations. The proposed DTM is a more powerful tool for future designs of CDMs in practical application systems.

## Design Principle of the Proposed Decreasing Thickness Model with Formulas

**Figure 8:**
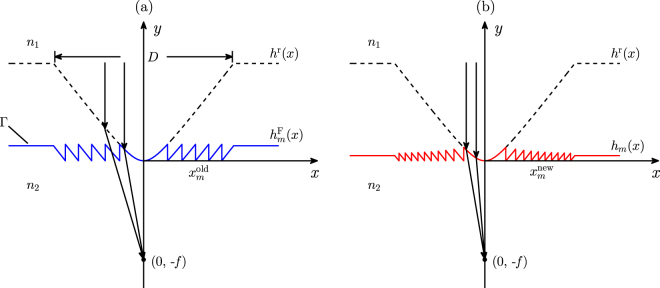
Schematics of the CDMs designed by (**a**) the ETM and (**b**) the DTM.

Before elaborating the proposed DTM, we firstly recall the design principle of the traditional ETM, as shown in Fig. 8(a). In Fig. 8(a), the black dashed curve represents the boundary of a cylindrical refractive microlens, which separates the whole space into two parts. The upper part is a dielectric with refractive index *n*_1_; the lower part is another dielectric with refractive index *n*_2_. The *xy* plane is the incident plane, and the CDM is infinite along the *z* axis. The CDM has an aperture size of *D*, which is symmetric about the *y* axis, as shown in Fig. 8(a). A plane wave with TE polarization is incident upon the refractive microlens boundary Γ, and it is assumed to be focused at (0, −*f*) owing to phase modulation of the microlens. The incident wavelength is *λ* in vacuum.

According to Fermat’s principle, the boundary height of the refractive microlens *h* ^r^(*x*) satisfies the following equation:

1 $${n}_{2}\sqrt{{x}^{2}+{[f+{h}^{{\rm{r}}}(x)]}^{2}}={n}_{1}{h}^{{\rm{r}}}(x)+{n}_{2}\,f,$$

where *x* is the transverse coordinate; *f* is the preset focal length of the CDM. After simplification, Eq. (1) can be written in an explicit form as follows^38^:

2 $${h}^{{\rm{r}}}(x)=\frac{{n}_{2}}{{n}_{1}^{2}-{n}_{2}^{2}}\{({n}_{2}-{n}_{1})f+\sqrt{{[({n}_{1}-{n}_{2})f]}^{2}+({n}_{1}^{2}-{n}_{2}^{2}){x}^{2}}\}.$$

In fact, the refractive microlens boundary in Eq. (2) is hyperbolic. As is well known, in wave optics an additional 2*π* phase does not change the optical element performance. The cylindrical refractive microlens can be thinned into a CDM by removing the abundant 2*π* phases. The obtained CDM is a traditional Fresnel lens, whose boundary height in the *m* th Fresnel zone is given by

3 $${h}_{m}^{{\rm{F}}}(x)=\,{\rm{Mod}}\,[{h}^{{\rm{r}}}(x),{\rm{\Delta }}h]={h}^{{\rm{r}}}(x)-| m| \,{\rm{\Delta }}h,\quad m=0,\pm 1,\pm 2,\pm 3,\ldots ,$$

where Mod[*A*, *B*] = *A* − Int[*A*/*B*] × *B* stands for the residual function; Int[*A*/*B*] represents the maximum integer not larger than *A*/*B*; *A* and *B* are positive real numbers; *m* = 0, ±1, ±2, ±3, … denotes the Fresnel zone ordinal number. The jump height Δ*h* corresponds to 2*π* phase modulation, i.e., we have 2*π*(*n*_1_ − *n*_2_)Δ*h*/*λ* = 2*π* or Δ*h* = *λ*/(*n*_1_ − *n*_2_). It is seen from Eq. (3) that the CDM has equal maximum thicknesses in all the Fresnel zones, as shown by the blue curve in Fig. 8(a). Accordingly, this design model is called as the ETM. Now we can determine the boundary jump points from the following equation

4 $${h}^{{\rm{r}}}({x}_{m}^{{\rm{old}}})=| m| \,{\rm{\Delta }}h,$$

where ${x}_{m}^{{\rm{old}}}$ represents the transverse coordinate of the jump points, as shown in Fig. 8(a). Eq. (4) can be simplified as

5 $${x}_{m}^{{\rm{old}}}=\pm \frac{1}{{n}_{2}}\sqrt{\frac{{m}^{2}{\lambda }^{2}({n}_{1}+{n}_{2})}{({n}_{1}-{n}_{2})}+2| m| \,{n}_{2}\lambda f},\quad m=0,\pm 1,\pm 2,\pm 3,\ldots $$

Although the ETM attenuates a refractive microlens into a CDM, it has two problems. Firstly, although the phase change is 2*π* in the vertical direction at the Fresnel zone edges, it deviates more or less in regard to the preset focal position. Secondly, since the segmented CDM boundary parts are moved from the refractive microlens, they no longer focus the incident plane wave on a same focal position. Two rectification strategies are proposed to overcome the two problems. One rectification is the boundary jump positions. In the new design model, the phase variation is exactly 2*π* with respect to the preset focal position at the Fresnel zone edges. Therefore, the boundary jump point of the *m* th Fresnel zone satisfies:

6 $${n}_{2}\sqrt{{({x}_{m}^{{\rm{new}}})}^{2}+{f}^{2}}-{n}_{2}f=| m| \,\lambda ,\quad m=0,\pm 1,\pm 2,\pm 3,\ldots $$

where ${x}_{m}^{{\rm{new}}}$ represents the new boundary jump positions, as shown in Fig. 8(b). On simplifying Eq. (6), we can write ${x}_{m}^{{\rm{new}}}$ as

7 $${x}_{m}^{{\rm{new}}}=\pm \frac{1}{{n}_{2}}\sqrt{{m}^{2}{\lambda }^{2}+2| m| \,{n}_{2}\lambda f},\quad m=0,\pm 1,\pm 2,\pm 3,\ldots $$

The other rectification is the boundary profile of the CDM, which is strictly deduced by Fermat’s principle. That is to say, two arbitrary light beams in the same Fresnel zone have the same optical path, and its mathematical expression is:

8 $${n}_{1}{h}^{m}(x)+{n}_{2}\sqrt{{({x}_{m}^{{\rm{new}}})}^{2}+{f}^{2}}={n}_{2}\sqrt{{x}^{2}+{[{h}_{m}(x)+f]}^{2}},$$

where *h*_*m*_(*x*) denotes the boundary height in the *m* th Fresnel zone of the CDM. By substituting Eq. (7) into Eq. (8), we can obtain the CDM boundary equation as

9 $$\begin{array}{ccc}{h}_{m}(x) & = & \frac{1}{{n}_{{\rm{1}}}^{2}-{n}_{{\rm{2}}}^{2}}\{({n}_{2}-{n}_{1}){n}_{2}f-{n}_{1}| m| \lambda +{n}_{2}\sqrt{{[({n}_{1}-{n}_{2})f-| m| \lambda ]}^{2}+({n}_{{\rm{1}}}^{2}-{n}_{{\rm{2}}}^{2}){x}^{2}}\},\\ \,m\, & = & 0,\pm 1,\pm 2,\pm 3,\ldots \end{array}$$

In Fig. 8(b), the red curve shows a schematic of the CDM boundary, whose maximum thickness decreases as the Fresnel zone ordinal number increases. Therefore, we call the new design model as the DTM.
